# The influence of college students' exercise participation on feelings of self-deficiency: the mediating effect of social support

**DOI:** 10.3389/fpsyg.2025.1560859

**Published:** 2025-05-22

**Authors:** Yunping Xia, Min Luo, Bo Liu, Yunxiang Fan, Kai Tang, Yong Tang, Xiang Zhang

**Affiliations:** ^1^College of Physical Education, Hunan Normal University, Changsha, China; ^2^Sports Department, Hunan Business Vocational and Technical College, Changsha, China; ^3^Sports Department, Yongzhou Vocational Technical College, Yongzhou, China; ^4^Sports Department, Hunan University of Humanities, Science and Technology, Loudi, China

**Keywords:** college students, exercise participation, social support, self-deficiency, mental health

## Abstract

**Objective:**

To explore the influence of college students' exercise participation on their feelings of self-deficiency and its mechanisms, with social support as a mediating variable.

**Methods:**

The study utilized the Physical Activity Rating Scale (PARS-3), the Feelings of Inadequacy Scale (FIS), and the Social Support Rating Scale (SSRS). A stratified random sampling method was used to select 981 college students from different provinces and universities for a questionnaire survey. Statistical methods, including confirmatory factor analysis, regression analysis, and the Bootstrap method, were employed for data analysis.

**Results:**

Exercise participation significantly and negatively predicted college students' feelings of self-deficiency. Social support exhibited a significant mediating effect between exercise participation and self-deficiency. Furthermore, the three dimensions of social support—subjective support, objective support, and support utilization—showed significant indirect effects. The mediation pathways were as follows: exercise participation → subjective support → self-deficiency, exercise participation → objective support → self-deficiency, and exercise participation → support utilization → self-deficiency.

**Conclusion:**

Exercise participation significantly reduces feelings of self-deficiency among college students, either directly or indirectly through social support. In the future, universities can help mitigate students' self-deficiency and promote their physical and mental wellbeing by optimizing physical activity spaces and constructing social support networks from the perspectives of exercise participation and social support.

## 1 Introduction

In today's fast-paced and highly competitive society, college students often face significant pressures arising from academic workloads, career advancement, and increasingly complex social relationships (Li and Gao, 2024). These intertwined sources of stress serve as potential factors triggering psychological issues among college students. In particular, when confronted with challenges and difficulties, students may question and negate their self-worth and abilities, leading to negative self-perceptions. This, in turn, manifests as heightened feelings of low mood, inferiority, and self-deficiency, potentially escalating into severe mental health issues such as anxiety and depression.

Self-deficiency sentiment (SSD) refers to a subjective psychological state where college students perceive or are aware of deficiencies, inadequacies, or inferiority in certain aspects of themselves. SSD encompasses five dimensions: social confidence, learning ability, self-esteem, appearance, and physical fitness (Bernichon et al., 2003). Beyond objective recognition of actual deficiencies, SSD involves excessive self-criticism, undervaluation, and an exaggerated focus on perceived flaws. The causes may stem from biased evaluations of personal abilities, inappropriate comparisons with peers or societal standards, and prolonged internalization of negative feedback. Excessive SSD can distort students' perceptions of their abilities and value, resulting in feelings of inferiority, anxiety, and depression, which negatively impact academic performance and social behaviors (Zhuang et al., 2021).

Exercise participation refers to individuals' attitudes and behaviors in actively engaging in physical activities, which positively influence physical fitness, mental health, and social adaptability (Teixeira et al., 2012). According to the Health Promotion Theory (Kim and Yoo, 2024), engaging in physical activities not only enhances physical fitness and reduces disease risks but also alleviates stress, depression, and anxiety, thereby promoting mental health. Additionally, it improves social skills, interpersonal relationships, and self-worth. Such positive attitudes and behaviors can mitigate feelings of self-deficiency arising from cognitive distortions and social inadequacies. Empirical studies also demonstrate (Karunagharan, 2023; Joldasbayev, 2024; Skodlar et al., 2013) that exercise participation significantly improves individuals' sense of belonging, psychological resilience, mental and physical health, interpersonal relationship quality, quality of life, self-esteem, and self-efficacy, while reducing negative outcomes such as aggression, work stress, anxiety, and self-deficiency. Therefore, this study hypothesizes H1: Exercise participation significantly and negatively predicts self-deficiency.

Social support refers to the various resources and assistance individuals receive from their social networks that alleviate psychological stress, ease tension, and enhance social adaptability. It comprises three dimensions: objective support, subjective support, and support utilization (Inui et al., 2022). Previous studies suggest (Che et al., 2018) that the mechanism by which social support enhances health primarily relies on two theoretical models: the “buffering” model and the “main effect” model. The buffering model posits that social support mitigates or eliminates the adverse effects of stressors on physical and mental health by providing a buffering effect during stressful or psychologically taxing events. The main effect model, on the other hand, asserts that social support has an inherently positive impact independent of stressors, promoting physical and mental health regardless of the presence of stressful circumstances. Empirical evidence (Wijaya et al., 2024; Teng, 2024; Tan et al., 2022) highlights the significant correlation between social support and mental health. Social support not only enhances self-esteem, self-efficacy, resilience, and psychological flexibility but also alleviates the negative impacts of depression, non-suicidal self-injury, psychological fatigue, and negative emotions on mental health (Naxton, 2024; Slesinger, 2021; Ng, 2024). Moreover, studies (Kelly et al., 1991) confirm that positive health beliefs and exercise behaviors significantly predict higher levels of social support. Based on these findings, this study predicts that exercise participation may reduce college students' self-deficiency by enhancing social support. Hence, hypothesis H2 is proposed: Social support mediates the relationship between exercise participation and self-deficiency.

Although preliminary research has explored the relationship between physical exercise and self-deficiency (Chu, 2022), such as Yin's (2023) findings that exercise participation provides college students with a platform to showcase and challenge themselves, fostering a sense of achievement and confidence that contributes to positive self-perceptions, self-efficacy, and psychological resilience, the mechanisms underlying the impact of exercise participation on self-deficiency remain unclear. Zhang et al. (2022) noted that exercise participation significantly regulates the psychological resilience of university faculty and indirectly influences occupational burnout through this mediating factor. However, how exercise participation influences self-deficiency and its underlying mechanisms require further exploration. Therefore, this study investigates the mediating role of social support in the relationship between exercise participation and self-deficiency among college students. By delving into the specific pathways of influence, this research aims to clarify the multifaceted value of exercise participation and provide scientific evidence and practical guidance for preventing and mitigating psychological deficiencies in college students, thereby fostering their holistic development.

## 2 Research methods

### 2.1 Data collection method

The data for this study were collected through an online questionnaire distributed *via* Wenjuanxing, a popular survey platform in China. The survey was conducted anonymously, and participants were fully informed about the study's purpose and provided informed consent prior to completing the questionnaires. The data collection process was standardized, and all participants filled out the survey independently, which minimized biases that may arise from face-to-face interactions (social desirability bias or researcher influence). This study has obtained ethical approval from the Research Ethics Committee of Hunan Normal University, with the approval number of 20250304.

### 2.2 Research sample

A stratified random sampling method was used to select Guangdong Province (eastern region), Hunan Province (central region), and Sichuan Province (western region) as sample provinces, based on differences in geographic location and economic development levels (Table 1). Within each province, one double first-class/elite undergraduate university, one regular undergraduate university, and one vocational college were randomly selected as sample units, according to educational tiers. From each sample unit, 2–4 classes of students across different grades were randomly chosen as participants. After informing participants of the study purpose and obtaining informed consent, 981 valid data samples were collected, including 533 males (54.3%) and 448 females (45.7%); 318 freshmen (32.4%), 303 sophomores (30.9%), 198 juniors (20.2%), and 162 seniors (16.5%); 366 students from double first-class/elite universities (37.3%), 315 from regular undergraduate universities (32.1%), and 300 from vocational colleges (30.6%); 201 only children (20.5%) and 780 non-only children (79.5%); 138 urban household registrations (14.1%), 333 town/county household registrations (34.0%), and 510 rural household registrations (51.9%).

**Table 1:** Demographic characteristics of the study sample.

**Variable**	**Category**	**Number (*n*)**	**Percentage (%)**
Gender	Male	533	54.3
	Female	448	45.7
Grade Level	Freshman	318	32.4
	Sophomore	303	30.9
	Junior	198	20.2
	Senior	162	16.5
School Type	Double First-Class/Elite University	366	37.3
	Regular Undergraduate University	315	32.1
	Vocational College	300	30.6
Only-Child Status	Only Child	201	20.5
	Non-Only Child	780	79.5
Household Registration	Urban	138	14.1
	Town/County	333	34
	Rural	510	51.9

### 2.3 Measurement tools

#### 2.3.1 Exercise participation

The Physical Activity Rating Scale (PARS-3) revised by Liang Deqing (Liang, 1994) was used to measure exercise participation. The scale evaluates participants' exercise levels based on three dimensions: duration, intensity, and frequency of participation, with the formula: exercise level = intensity × duration × frequency. The scale includes four items scored on a 5-point Likert scale. Intensity and frequency are scored from 1 to 5, while duration is scored from 0 to 4. The total score ranges from 0 to 100, with higher scores indicating greater exercise participation. In this study, the Cronbach's Alpha coefficient for the scale was 0.871, and the Spearman-Brown split-half reliability was 0.804.

#### 2.3.2 Classification of exercise participation

In this study, exercise participation was measured using the Physical Activity Rating Scale (PARS-3), which assesses three dimensions: frequency, intensity, and duration of exercise. Participants were classified based on the following criteria: low frequency (less than once per week), moderate frequency (1–3 times per week), and high frequency (more than three times per week); low intensity (light activities, such as walking), moderate intensity (moderate activities, such as jogging), and high intensity (vigorous activities, such as running); exercise duration was categorized as less than 30 minutes, 30–60 min, and more than 60 min. This classification method allows for a more detailed analysis of participants with varying levels of exercise involvement.

#### 2.3.3 Self-deficiency sentiment

The Self-Deficiency Sentiment Scale (FIS) developed by Fleming and Courtney (Buss, 2001) was used to assess participants' self-deficiency. The scale includes five dimensions: social confidence, learning ability, self-esteem, appearance, and physical fitness, with 36 items scored on a 7-point Likert scale ranging from 1 (“never”) to 7 (“always”). Higher scores indicate stronger self-deficiency sentiments. In this study, the Cronbach's Alpha coefficient for the scale was 0.897, and the Spearman-Brown split-half reliability was 0.839.

#### 2.3.4 Social support

The Social Support Rating Scale (SSRS) developed by Xiao Shuiyuan (Xiao, 1994) was used to measure social support. This scale consists of 10 items across three dimensions: objective support, subjective support, and support utilization. Items 6 and 7 are scored based on the number of sources reported by participants (0 if no source), while the remaining items use a 4-point grading system (A, B, C, D) scored from 1 to 4. Higher total scores indicate greater levels of social support. In this study, the Cronbach's Alpha coefficient for the scale was 0.782, and the Spearman-Brown split-half reliability was 0.767.

### 2.4 Control variables

Given the significant effects of individual differences such as gender (Ackerman et al., 2001) and grade level (Lippa, 1995) on college students' self-deficiency, this study included key demographic factors—gender, grade level, only-child status, type of school, and household registration—as control variables. This was done to eliminate interference from individual differences and ensure the accuracy and validity of the research results, thus providing a clearer understanding of the mechanisms through which exercise participation affects self-deficiency.

### 2.5 Data processing

The collected data were systematically processed and analyzed using software tools such as SPSS 26.0, AMOS 21.0, Excel 2024, and Mplus 8.3. The internal consistency and stability of the scales were tested using Cronbach's Alpha coefficients and Spearman-Brown split-half reliability. Confirmatory factor analysis was employed to check for potential common method bias among the variables of exercise participation, self-deficiency, and social support. Descriptive statistics and correlation analyses were conducted to assess the status and interrelationships of the three variables. Hierarchical multiple regression analysis was used to verify the mediating role of social support in the relationship between exercise participation and self-deficiency. Path analysis and model fit testing using Mplus 8.3 were performed to evaluate the goodness of fit of the influence pathway model.

## 3 Result

### 3.1 Control and testing of common method bias

#### 3.1.1 Procedural control

To enhance data quality, strict procedural controls were implemented in the research design and measurement process. A pilot survey was conducted to comprehensively review and revise the questionnaire items, ensuring clarity and accuracy. The sequence of questionnaire items was balanced to minimize order effects on the results. An anonymous survey approach was used to reduce social desirability bias. Measurements of the three variables—exercise participation, self-deficiency, and social support—were conducted at two separate time points to avoid confounding effects from overlapping measurement periods. Surveyors were uniformly trained to ensure the standardization and consistency of the data collection process. Additionally, trap questions and patterns of response regularity were employed to screen and clean the collected data, excluding invalid or anomalous responses.

#### 3.1.2 Statistical testing

Confirmatory factor analysis (CFA) was applied to test for common method bias between the variables of social support and self-deficiency. The dimensions of intensity, duration, and frequency in the Physical Activity Rating Scale (PARS-3) are independent and non-aggregable single indicators, and thus, common method bias testing was not required. The core of the CFA method involves testing a hypothesized single-factor model to evaluate whether “all data variation can be attributed to a single latent factor.” This approach assesses both convergent validity (whether measurement items effectively reflect the same construct) and discriminant validity (whether different constructs can be effectively distinguished).

The results showed that for the Self-Deficiency Sentiment Scale (FIS), χ^2^/df was 2.304, AGFI = 0.910, NFI = 0.906, CFI = 0.924, and RMSEA = 0.04. For the Social Support Rating Scale (SSRS), χ^2^/df was 2.209, AGFI = 0.905, NFI = 0.907, CFI = 0.903, and RMSEA = 0.05. These results indicate satisfactory levels of convergent and discriminant validity for the two variables, with no significant common method bias.

### 3.2 Descriptive statistics and correlation analysis

Table 2 shows that the mean score for exercise participation among college students was 31.532 ± 6.580, indicating a moderate level of participation based on the scoring standard (20–42 represents moderate activity levels). The mean score for self-deficiency sentiment was 110.388 ± 41.582, below the theoretical midpoint of 144, suggesting a generally low level of self-deficiency. The mean score for social support was 36.508 ± 6.951, above the theoretical midpoint of 24.5, indicating a relatively high level of social support. Correlation analysis revealed significant negative correlations between exercise participation and self-deficiency (*r* = −0.404, *P* < 0.05), significant positive correlations between exercise participation and social support (*r* = 0.243, *P* < 0.05), and significant negative correlations between social support and self-deficiency (*r* = −0.235, *P* < 0.01).

**Table 2:** Descriptive statistics and their correlation coefficients of sports participation, sense of self-deficiency, and social support among university students.

**Variables**	***M***	**SD**	**Physical activity**	**Self-perceived deficiency**	**Social support**
Physical activity participation	31.532	6.580	1.000		
Self-perceived deficiency	110.388	41.582	−0.404^*^	1.000	
Social support	36.508	6.951	0.243^*^	−0.235^**^	1.000

^*^p < 0.05, ^**^p < 0.01.

Additionally, demographic variable analysis showed significant differences in self-deficiency levels. Female students reported higher self-deficiency than male students (*T* = −2.383, *P* = 0.017), potentially due to gender differences in social confidence and physical self-perception influenced by societal expectations. Urban and town-dwelling students had significantly lower self-deficiency levels compared to rural students (*F* = 2.995, *P* = 0.049), possibly due to disparities in educational resources and socioeconomic environments, which affect confidence in learning abilities and self-esteem. No significant differences were observed for grade level, only-child status, or school type (*P* > 0.05).

### 3.3 Regression analysis of exercise participation on self-deficiency

A hierarchical regression analysis was conducted, with self-deficiency as the dependent variable. Gender, grade level, only-child status, school type, and household registration were included as independent variables, with categorical variables dummy-coded prior to regression. Results (Table 3) indicated that gender and household registration significantly and negatively predicted self-deficiency (*P* < 0.05). These demographic variables were subsequently controlled for in the analysis.

**Table 3:** Regression analyses of sport participation on feelings of self-deficiency.

**Dependent variable**	**Value**	**Gender**	**Grade**	**Yes/no**	**Only child**	**School type**	**Household registration location**	***F***	***R* ^2^**	**Δ*R*^2^**
Sense of self-deficiency	β	−0.171	0.434	0.035	−0.052	−0.060	−0.402	37.880^**^	0.163	0.161
	*t*	−5.240^**^	1.207	1.089	−1.637	−1.785^*^	−13.695^**^			

^*^P < 0.05.

^**^P < 0.01.

When exercise participation was added as an independent variable with demographic variables controlled, results showed that exercise participation significantly and negatively predicted self-deficiency (β = −0.402, *P* < 0.01), explaining 16% of the variance. Thus, hypothesis H1, stating that exercise participation significantly and negatively predicts self-deficiency, was supported.

### 3.4 Mediation effect of social support between exercise participation and self-deficiency

The bias-corrected percentile Bootstrap method was used to test mediation effects, as it provides higher stability and accuracy than the traditional Sobel test (Karazsia, 2009). This method involves repeated random sampling with replacement from the original dataset to generate multiple bootstrap samples, allowing the estimation of population parameters based on these sample statistics. The analysis employed Model 4 in the Process macro, with 5,000 bootstrap samples. A mediation effect is considered significant if the 95% confidence interval does not include zero. Gender, grade level, and other demographic variables were controlled to focus on the core relationship among variables.

Regression analysis (Table 4) showed that exercise participation positively predicted overall social support (β = 0.244, *P* < 0.01) and its dimensions. Exercise participation also negatively predicted self-deficiency (β = −0.265, *P* < 0.01). Social support and its dimensions—subjective support (β = −0.142, *P* < 0.01), objective support (β = −0.200, *P* < 0.01), and support utilization (β = −0.303, *P* < 0.01)—significantly and negatively predicted self-deficiency.

**Table 4:** Regression analyses for tests of the mediating effect of social support between sport participation and feelings of self-deficiency.

**Regression equation**		**Overall fit index**			**Significance of regression coefficients**	
**Outcome variable**	**Predictor variable**	***R***	***R** ^2^*	***F***	β	***t***
Social support	Physical activity participation	0.243	0.059	12.142^**^	0.244	5.019^**^
Subjective support		0.316	0.100	12.470	0.259	4.411^**^
Objective support		0.220	0.048	6.744	0.149	2.304^*^
Support utilization		0.304	0.092	12.141	0.235	5.197^**^
Sense of self-deficiency	Physical activity participation	0.402	0.161	9.421^**^	−0.265	−5.103^**^
	Social support	0.235	0.055	8.142^**^	−0.270	−6.121^**^
	Subjective support	0.100	0.010	4.101^**^	−0.142	−2.012^*^
	Objective support	0.241	0.058	9.690^**^	−0.200	−3.643^**^
	Support utilization	0.192	0.037	6.372^**^	−0.303	−5.144^**^

All variables were standardized before being entered into the regression equation.

^*^P < 0.05.

^**^P < 0.01.

Mediation analysis results (Table 5) showed that the direct effect of exercise participation on self-deficiency was significant [β = −0.402, Bootstrap 95% CI (−0.520, −0.331), excluding zero]. Social support exerted a total indirect effect [β = −0.189, Bootstrap 95% CI (−0.196, −0.165), excluding zero], indicating a significant mediating role of social support. This mediation effect comprised three specific pathways:

1. Exercise participation → Subjective support → Self-deficiency [effect size: −0.029, Bootstrap 95% CI (−0.148, −0.030), excluding zero], confirming the mediating role of subjective support.
2. Exercise participation → Objective support → Self-deficiency [effect size: −0.061, Bootstrap 95% CI (−0.066, −0.117), excluding zero], confirming the mediating role of objective support.
3. Exercise participation → Support utilization → Self-deficiency [effect size: −0.099, Bootstrap 95% CI (−0.120, −0.097), excluding zero], confirming the mediating role of support utilization.

**Table 5:** Bootstrap analyses of tests of the mediating effect of social support between sport participation and feelings of self-deficiency.

**Impact pathways**	**Standardized effect value**	**Proportion of total effect**	**Bootstrapped standard error**	**95% CI**		**Significance**
				**Upper limit**	**Lower limit**	
Total effect	−0.591^**^	—	0.012	−0.425	−0.480	Significant
Direct effect	−0.402^**^	68.02%	0.043	−0.520	−0.331	Significant
Total indirect effect	−0.189^**^	31.98%	0.041	−0.196	−0.165	Significant
Physical activity participation → subjective support → sense of self-deficiency	−0.029^*^	4.91%	0.005	−0.148	−0.030	Significant
Physical activity participation → objective support → sense of self-deficiency	−0.061^**^	10.32%	0.012	−0.066	−0.117	Significant
Physical activity participation → support utilization → sense of self-deficiency	−0.099^**^	16.75%	0.045	−0.120	−0.097	Significant

**Figure 1:**
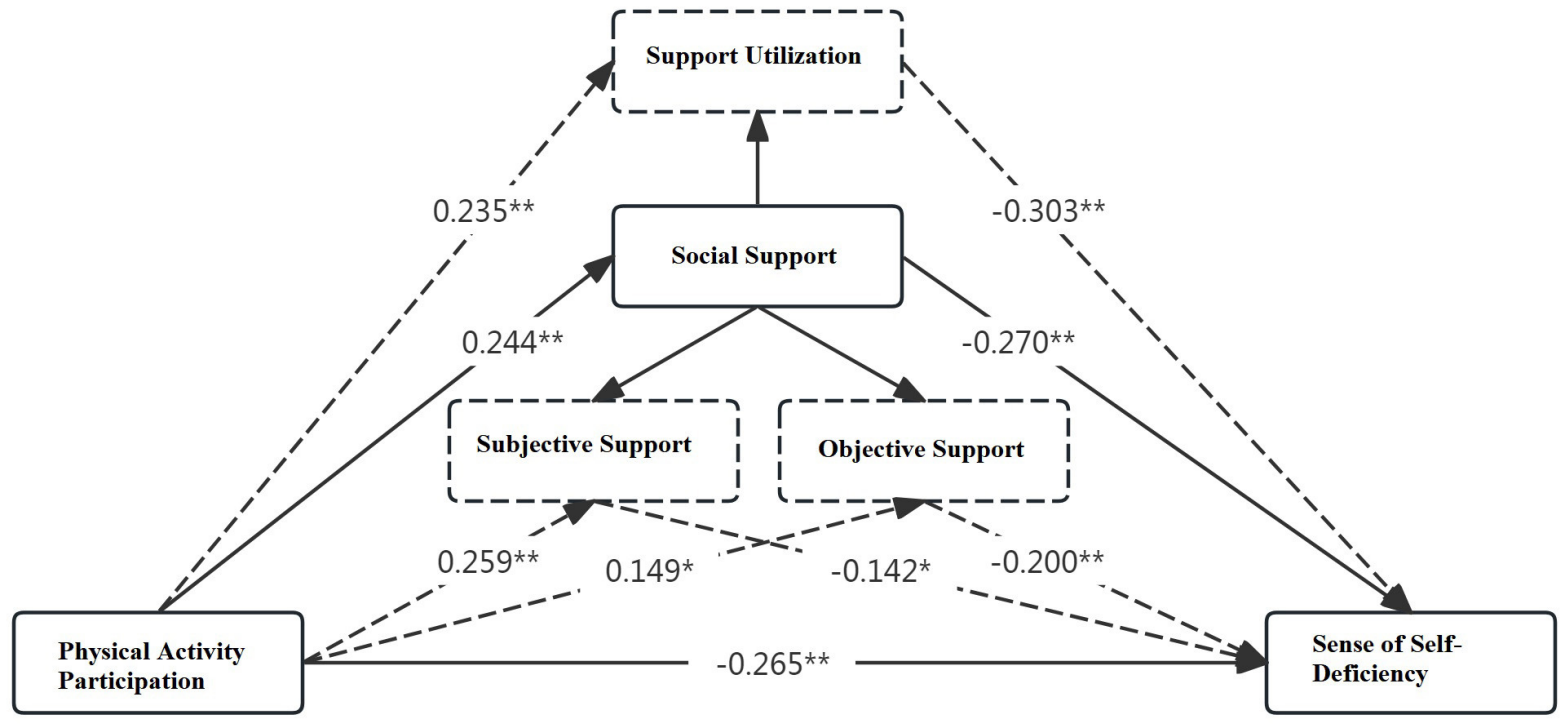
Results of structural equation modeling analysis.

Model fit testing using Mplus 8.3 indicated good fit indices: χ^2^/df = 2.410, RMSEA = 0.089, CFI = 0.903, TLI = 0.905, SRMR = 0.059. Structural equation modeling (Figure 1) demonstrated that exercise participation positively influenced social support (β = 0.244, *P* < 0.01), and social support negatively influenced self-deficiency (β = −0.270, *P* < 0.01). These results validated the mediating role of social support and its dimensions—subjective support, objective support, and support utilization—in the relationship between exercise participation and self-deficiency, supporting hypothesis H2.

## 4 Discussion

### 4.1 Comparison with previous studies

This study found that exercise participation significantly reduced college students' sense of self-deficiency, a result consistent with previous research (Kotera et al., 2022; Petrocchi and Couyoumdjian, 2016; Schanche et al., 2021). Numerous international studies have shown that physical exercise can reduce self-deficiency by enhancing self-efficacy and self-confidence (Jones et al., 2005; Yang et al., 2023). However, unlike Western studies, which primarily focus on improving self-efficacy and independence, our research found that, within the Chinese cultural context, social support plays an important mediating role in the relationship between exercise and self-deficiency. This finding supports the significant role of social networks in individual mental health within collectivist cultures, highlighting the positive impact of family, peer, and school support (Chang and Chen, 2022). Furthermore, this study revealed that gender and household registration background significantly affect self-deficiency, with females and rural students exhibiting higher levels of self-deficiency, which is consistent with previous studies (Zhang, 2020; Sagar and Jowett, 2012). These differences may be related to societal expectations of different groups and differences in self-perception within the cultural context, particularly when social pressure regarding body shape and appearance is more pronounced, which can affect the self-evaluation of females and rural students.

### 4.2 Direct effect of exercise participation on self-deficiency

College students, being in a critical transitional period from campus to society, face complex and dynamic social environments and challenges. These include high expectations and pressures from families, structural employment difficulties, and self-negation triggered by peer comparisons (Zhang and Nukulkij, 2024). Such factors make them prone to feelings of self-deficiency, which can negatively affect their learning motivation, social interactions, emotional regulation, behavior patterns, and self-confidence. These issues may even escalate into severe psychological problems, making self-deficiency an urgent mental health concern.

According to the Health Promotion Theory, this study hypothesized that exercise participation significantly and negatively predicts self-deficiency. The empirical results not only confirmed this hypothesis but also aligned with previous findings by Soyeon A (Soyeon and Fedewa, 2011), Mingli (Liu et al., 2015), and Wu Jingtao (Wu et al., 2022), which demonstrated that physical exercise significantly alleviates negative emotions and promotes mental health. The direct mechanisms through which exercise participation reduces self-deficiency in college students can be understood across psychological, behavioral, and social dimensions:

#### 4.2.1 Psychological dimension

Exercise participation reduces self-deficiency by enhancing physical self-esteem, self-efficacy, and emotional regulation. Research indicates (Strahan et al., 2006) that societal aesthetic standards significantly influence college students' body image perceptions, leading many students to underestimate their physical attractiveness relative to societal norms. This misperception affects self-concept, emotional experiences, and strategies for weight management, potentially fostering low self-esteem and self-deficiency. However, regular exercise participation induces positive psychological changes, such as increased release of neurotransmitters like endorphins, which evoke feelings of pleasure, excitement, and satisfaction. These positive emotional experiences improve students' emotional states, resilience, and ability to cope with adversity. By fostering a more positive body image and reinforcing self-recognition, exercise participation helps improve self-esteem and reduces tendencies toward self-deprecation and depression stemming from physical appearance or fitness deficiencies (Wang et al., 2024).

#### 4.2.2 Behavioral dimension

Consistent and regular exercise participation enhances self-regulation and promotes the internalization of positive behavior patterns, thereby weakening perceptions of self-deficiency. Despite facing challenges such as academic stress, emotional relationships, diverse social activities, and digital device dependence (Marciano et al., 2022), understanding the intrinsic value of exercise—such as health benefits, psychological enhancement, and social functions—can motivate students to participate autonomously. Over time, this fosters the integration of exercise into their lifestyles as a stable behavioral pattern (Kumanyika et al., 2008). Such patterns strengthen their sense of control and accomplishment, reduce self-doubt and self-negation, and promote positive self-perceptions. Particularly in team sports, the experience of successfully completing tasks or competitions enhances confidence, reinforces self-efficacy, and fosters positive behavioral tendencies.

#### 4.2.3 Social dimension

Exercise participation provides an effective platform for social interaction, helping students enhance social skills, build social confidence, and reduce self-deficiency. During sports activities, students engage in close communication and collaboration with teammates to achieve shared goals. This process promotes effective interaction in diverse environments, enhances teamwork abilities, and broadens social networks by interacting with individuals from varied backgrounds. These experiences improve social adaptability, foster openness and inclusivity, and alleviate self-deficiency linked to social confidence. However, attention should be paid to students who invest significant effort in sports but fail to achieve desired outcomes, to prevent feelings of learned helplessness (Fox and Magnus, 2014).

### 4.3 Mediating role of social support between exercise participation and self-deficiency

Mediation analysis confirmed that social support significantly mediates the relationship between exercise participation and self-deficiency, supporting hypothesis H2. The “buffering” and “main effect” theories of social support (Liu et al., 2021) highlight its protective role in promoting mental health. Unlike other groups, college students face diverse challenges in academics, employment, social relationships, and emotions, yet their access to social support networks is relatively limited, primarily relying on peers, teachers, counselors, and family. Exercise participation provides opportunities for broader interactions, expanding their social support networks, and enhancing their ability to perceive and utilize social support.

When students encounter challenges, the emotional support, encouragement, and practical problem-solving offered by sports team members form a robust support system. This system enhances self-efficacy through positive feedback, reduces perceived stress, and significantly shapes their self-perception. Not only does it directly improve psychological states and alleviate negative emotions, but it also fosters the accumulation of positive self-cognition over time, reducing self-deficiency levels.

The mediating effect of social support is comprised of three pathways:

1. Subjective support pathway:

Subjective support refers to the emotional support individuals feel, such as being respected, understood, cared for, and accepted. During sports participation, students often receive recognition and encouragement from peers, teammates, and coaches. These positive external feedbacks reinforce their subjective experiences of support, boosting confidence and motivation to engage in sports. This creates a virtuous cycle of positive psychology and behavior, reducing self-negation and mitigating self-deficiency caused by external pressures or internal self-doubt.

2. Objective support pathway:

Objective support involves tangible resources and assistance received during social interactions, such as material aid, stable social connections, and practical help. Through sports, students gain access to resources like participation opportunities, professional facilities, and funding provided by schools, families, and society. Additionally, sports broaden social networks, enabling stable relationships and friendships that offer valuable advice and guidance. These factors collectively reduce self-deficiency.

3. Support utilization pathway:

Support utilization reflects the ability to actively and effectively mobilize social support resources in times of stress or difficulty. Among the three pathways, support utilization showed the most significant mediating effect (Table 5), emphasizing its crucial role in reducing self-deficiency. Limited access to social support networks can leave students feeling isolated, especially when facing academic pressures, career uncertainties, or employment challenges (Van Hees et al., 2015). Exercise participation as a social activity helps expand support networks, strengthen communication with coaches, teammates, family, and friends, and provide timely practical and emotional support. This enhances students' ability to face challenges and fosters a sense of belonging and personal identity, thereby reducing self-deficiency.

Exercise participation significantly and negatively predicts self-deficiency among college students, highlighting its positive role in mitigating self-deficiency levels. The mediation model revealed that exercise participation impacts self-deficiency both directly and indirectly through social support, offering valuable insights into the mechanisms of this relationship. These findings emphasize the importance of encouraging exercise participation and fostering robust social support networks in addressing self-deficiency. Future research could further explore additional mediating or moderating variables to uncover the complex interplay of factors influencing self-deficiency. Such studies could help design more comprehensive interventions and strategies to promote exercise participation and strengthen social support systems, ultimately fostering the positive development of college students.

### 4.4 Research limitations

This study identified the mediating role of social support in the relationship between exercise participation and self-deficiency, clarifying its pathways of influence. However, several limitations remain:

1. The sample size was relatively limited due to time and resource constraints, making it difficult to comprehensively reflect the overall status of college students. Future research should expand and optimize the sample size and structure to enhance generalizability and validity.
2. The study used a cross-sectional survey, which effectively collected large samples but could not infer causal relationships between variables. Longitudinal designs, such as cross-lagged studies or experimental research, are needed to explore causal chains and mechanisms of influence.
3. While focusing on the mediating role of social support, other potential variables influencing self-deficiency, such as family environment, economic conditions, teacher-student relationships, and campus culture, were overlooked. Future studies should incorporate these factors to explore their mediating or moderating effects, providing a more comprehensive understanding of the complex mechanisms through which exercise participation impacts self-deficiency.
4. This study classified exercise participation using the PARS-3 scale; however, self-reported exercise participation may introduce biases, particularly in the classification of frequency and intensity, affecting the accuracy of the classification. The sample included fewer low-frequency and low-intensity exercisers, which may not fully reflect the impact of these groups. Future research could improve the classification method and ensure balanced analysis of participants at all levels of exercise involvement through more precise measurement tools or longitudinal tracking studies.

## 5 Conclusion

Exercise participation directly reduces self-deficiency among college students and exerts an additional indirect effect through the mediating role of social support. This dual pathway underscores the multifaceted benefits of exercise participation and highlights the potential of leveraging social support to enhance its impact. The findings provide a scientific basis for interventions aimed at reducing self-deficiency and promoting the physical and mental wellbeing of college students.

The study's results suggest targeted strategies for optimizing exercise participation and enhancing social support to effectively reduce self-deficiency. These strategies include optimizing sports activity spaces by upgrading campus facilities, fostering a robust campus sports culture, and establishing sports guidance services. Building social support networks is equally essential, involving the development of a comprehensive support system, promotion of practical activities such as volunteering and academic exchanges, and the creation of a collaborative framework connecting families, schools, and communities. These efforts aim to foster students' physical and mental wellbeing while reducing self-deficiency through enhanced engagement in sports and strengthened social support systems. This study proposes the following specific measures:

(1) Universities should optimize sports facilities and activity spaces, construct multi-functional and easily accessible sports facilities, and offer a variety of sports activities to encourage students to participate more actively in exercise.
(2) Strengthening the social support network, schools can promote student interaction and build a robust social support system by organizing group activities, providing psychological counseling programs, and enhancing teacher-student support.
(3) For students from different gender and regional backgrounds (female students and rural students), universities should develop personalized mental health and exercise programs to help them overcome feelings of self-deficiency.
(4) Schools can develop a comprehensive health promotion strategy by integrating physical exercise, mental health education, and social activities to foster the overall physical and mental development of students.

## Data Availability

The original contributions presented in the study are included in the article/supplementary material, further inquiries can be directed to the corresponding authors.
